# Editorial: Solution thermodynamics and dynamics in lithium-bearing brine systems

**DOI:** 10.3389/fchem.2024.1368600

**Published:** 2024-01-30

**Authors:** Yafei Guo, Lingzong Meng, Bamidele Victor Ayodele

**Affiliations:** ^1^ Key Laboratory of Marine Resource Chemistry and Food Technology (TUST), Ministry of Education, Tianjin Key Laboratory of Brine Chemical Engineering and Resource Eco-Utilization, College of Chemical Engineering and Materials Science, Tianjin University of Science and Technology, Tianjin, China; ^2^ School of Chemistry and Chemical Engineering, Linyi University, Linyi, China; ^3^ Chemical Engineering Department, Universiti Teknologi PETRONAS, Bandar Seri Iskandar, Perak, Malaysia; ^4^ CO_2_ Research Centre (CO_2_RES), Institute of Contaminant Management, Universiti Teknologi PETRONAS, Bandar Seri Iskandar, Perak, Malaysia

**Keywords:** Li-bearing brine systems, solution thermodynamics, solution structure and dynamics, phase equilibria, computational solution chemistry

The global demand for lithium has been steadily rising for several decades. Currently, lithium is predominantly utilized in batteries for mobile gadgets and electric cars. The increasing pace of lithium resource utilization and the depletion of solid mineral resources have led to a growing interest in the development of brine lithium resources. The brines have undergone long-term geological processes that have resulted in the concentration of several precious elements. The concentration and fractional crystallization of brine in solar evaporation ponds are essential procedures for extracting valuable elements. Hence, it is imperative to elucidate the chemical characteristics of these saline and lithium-bearing hypersaline systems in order to gain insight into and enhance industrial processes. The study of thermodynamics and dynamics in brine systems containing lithium can provide essential data on the thermodynamics and dynamics of the system. This data is crucial for understanding the chemical behaviour of brine and for effectively using lithium brine resources.

The recent advances in the removal of radioactive iodine by bismuth (Bi)-based materials were reported by Hao et al. Based on the extensive review, the adsorption capabilities of several Bi-based compounds exceeded those of commercially available adsorbents. The majority of Bi-based modified absorbents possess the benefits of a large specific surface area and a abundant amount of active sites. This highlights the feasibility of generating customized materials based on current supports using Bi as the main component. Characterization, leaching studies, and immobilization experiments demonstrate that Bi, Bi_2_O_3_, and Bi_2_S_3_ may undergo a reaction with iodine to produce Bi_x_O_y_I_z_. At elevated temperatures, the stability of Bi_5_O_7_I surpasses that of BiOI and BiI_3_, indicating that chemisorbed iodine can be liberated, and more bismuth is required for immobilization. Despite the abundance of Bi-based products, there is a dearth of comprehensive study and comparison.

Liu et al. reported the hydration of Li^+^ and Mg^2+^ in subnano carbon nanotubes using a multiscale theoretical approach. The findings indicate that the 0.60 nm CNTs have a stronger confinement impact on Li^+^ ions, making it challenging for Cl^−^ ions to be involved in the production of the initial coordination shell of Li^+^. This is due to the establishment of a charge-induced polarisation (CIP) between Li^+^ and Cl^−^ ions, which occurs through an intermediary step involving [Li(H_2_O)_4_]^+^. However, the development of charge-transfer interaction (CIP) between lithium ions (Li^+^) and chloride ions (Cl^−^) is more favourable in the remaining four carbon nanotubes (CNTs). In these cases, the lithium ions are coordinated with three water molecules ([Li(H_2_O)_3_]^+^Cl^−^), resulting in a very stable complex.

The modeling of bio-hydrogen recovery from agro-industrial wastewater was reported by Hossain et al. (2022). The authors employed data-driven machine learning algorithms to model biohydrogen production from dairy, chicken processing, and palm oil mills. The production of biohydrogen from different wastewaters may be adjusted in real-time with the aid of machine learning algorithms, increasing process effectiveness and effective usage of resources like energy and materials. The historical data from the processes may be used to optimize intended products and constantly enhance process performance. Natural gas as a clean energy source has been anticipated to play a vital role in transitioning to net-zero emissions by 2050. With increasingly rigorous environmental regulations, liquified natural gas (LNG) is seen as a possible alternative fuel for the maritime sector, although the safety issues brought on by LNG leakage incidents need to be taken into consideration and studied. Wang et al. (2022) employed computational fluid dynamics to simulate cryogenic safety analysis in an LNG-powered ship during leakage. When LNG leaked, the range of the cryogenic area in the fuel gas preparation room was correlated with the direction of the flow field. Traditional energy sources not only have finite supplies but also release harmful byproducts into the environment. One of the most stimulating eco-friendly energy sources is the photovoltaic (PV) solar system. Srivastava et al. (2022) evaluated a grid-connected solar-powered microgrid in northern India using PV*Syst software. Rooftop solar panels are a technologically and economically viable way to provide power in northern India, as shown by the comparative analysis. Vennila et al. (2022) reported static and dynamic mixed economic and emission dispatch problem that was solved using the tournament selection-based ALO algorithm. According to the findings, the production of clean energy can be stabilized in the future by combining a hybrid dynamic economic and emission dispatch model with thermal power plants, wind turbines, solar panels, and energy storage devices to achieve a balance between operating costs and pollutant emissions. Prakash et al. (2022) presented a review of battery energy storage technologies for supplementary services in distribution grids. The review analysis includes a cost-benefit analysis and a list of energy storage laws as well as battery energy storage initiatives from across the world. Future Research Topics are also discussed, along with recommendations on how to overcome the implementation issues that come with integrating battery energy storage systems in distribution grids. The interest in modeling and optimization of clean energy processes is evidenced by the subject set’s cumulative view count, which at the time of writing this editorial had reached 4,746. There had also been 524 downloads of the different articles.

